# The life in sight application study (LISA): design of a randomized controlled trial to assess the role of an assisted structured reflection on life events and ultimate life goals to improve quality of life of cancer patients

**DOI:** 10.1186/1471-2407-13-360

**Published:** 2013-07-26

**Authors:** Renske Kruizinga, Michael Scherer-Rath, Johannes BAM Schilderman, Mirjam AG Sprangers, Hanneke WM Van Laarhoven

**Affiliations:** 1 Department of Medical Oncology, Academic Medical Center, University of Amsterdam, Meibergdreef 9, 1105, AZ Amsterdam, The Netherlands; 2 Faculty of Philosophy, Theology and Religious Studies, Radboud University Nijmegen, Erasmusplein 1, 6500, HD Nijmegen, The Netherlands; 3 Department of Medical Psychology, Academic Medical Center, University of Amsterdam, Meibergdreef 15, 1105, AZ Amsterdam, Netherlands

**Keywords:** Spiritual care, Quality of life, Meaning, Ultimate life goals, Palliative care, Contingency, Cancer patients, Spiritual wellbeing, Empowerment

## Abstract

**Background:**

It is widely recognized that spiritual care plays an important role in physical and psychosocial well-being of cancer patients, but there is little evidence based research on the effects of spiritual care. We will conduct a randomized controlled trial on spiritual care using a brief structured interview scheme supported by an e-application. The aim is to examine whether an assisted reflection on life events and ultimate life goals can improve quality of life of cancer patients.

**Methods/Design:**

Based on the findings of our previous research, we have developed a brief interview model that allows spiritual counsellors to explore, explicate and discuss life events and ultimate life goals with cancer patients. To support the interview, we created an e-application for a PC or tablet. To examine whether this assisted reflection improves quality of life we will conduct a randomized trial. Patients with advanced cancer not amenable to curative treatment options will be randomized to either the intervention or the control group. The intervention group will have two consultations with a spiritual counsellor using the interview scheme supported by the e-application. The control group will receive care as usual. At baseline and one and three months after randomization all patients fill out questionnaires regarding quality of life, spiritual wellbeing, empowerment, satisfaction with life, anxiety and depression and health care consumption.

**Discussion:**

Having insight into one’s ultimate life goals may help integrating a life event such as cancer into one’s life story. This is the first randomized controlled trial to evaluate the role of an assisted structured reflection on ultimate life goals to improve patients’ quality of life and spiritual well being. The intervention is brief and based on concepts and skills that spiritual counsellors are familiar with, it can be easily implemented in routine patient care and incorporated in guidelines on spiritual care.

**Trial registration:**

The study is registered at ClinicalTrials.gov: NCT01830075

## Background

Spirituality is increasingly recognized as an important domain to include in the care for patients with a life threatening illness [1-5]. In a recent Consensus Conference, spirituality has been defined as the aspect of humanity that refers to the way individuals seek and express meaning and purpose and the way they experience their connectedness to the moment, to self, to others, to nature, and to the significant or sacred [4]. According to reports in the United States and Canada, 50%-90% of cancer patients view religion or spirituality as personally important [6-8]. Religion and spirituality can offer a source of comfort, meaning, control and personal growth to patients who are confronted with a life-threatening disease [9,10]. Spirituality may be especially relevant for patients’ well-being. In a recent systematic review on the relationship between spirituality and well-being in cancer patients, the majority of identified studies observed a positive association between spirituality and well-being [11].

Recommendations from the 2005 National Consensus Project on Quality Palliative Care called for increased efforts to understand patients’ existential needs and to conduct and evaluate interventions to address these concerns [4]. Nevertheless, appropriate, effective, and brief interventions to address spiritual concerns are still lacking. One of the key-elements in these spiritual concerns is the experience of contingency: the experience that something is neither a necessity, nor an impossibility, everything could have been different [12]. Contingency will be experienced when it is problematic to incorporate an event into one’s story of life. The diagnosis of advanced cancer may be such an event. The aim of our study is to examine whether an assisted reflection on contingent life events and ultimate life goals can improve cancer patients’ quality of life.

### The experience of contingency

Cancer patients are confronted with a diagnosis and subsequent treatment that may have a large impact on their life perspective [13,14]. Their life lines are suddenly disrupted, which necessitates a reinterpretation of their lives. This experience is called experience of contingency [12]. Experiences of contingency prompt people to shape a meaningful relation to the situations they are confronted with. Meaningful implies: acting in such a way that it logically and plausibly connects to one’s actions in the past, as well as to desires, wishes and needs for the future [15,16]. In a traditional society, the contingency of action and choice was limited [13], but nowadays people have become individuals with their own personal, biographical story that they have to construct and justify by themselves [17,18]. They increasingly feel obliged to shape their own framework of interpretation for situations they are confronted with. Shaping such a framework of interpretation can be facilitated by the construction of a narrative [19,20]. A narrative configures separate events into an intelligible whole [20]. It creates a temporal coherence whereby a so-called plot links past, present and future to one another and to the personal goals that people pursue. In confrontation with a contingent situation an extra narrative effort is required to construct a new framework of interpretation which fits with one’s ultimate life goals [21].

### Ultimate life goals

In the way people react to the experience of contingency and the stories people tell about their life events, we can decipher the underlying life goals [22]. Personal goals express what people find really important. They are the intrinsic source of human action [20,23]. A distinction can be made between instrumental and ultimate life goals [24]. Instrumental goals refer directly to actions and the way actions are carried out, whereas more abstract goals provide information on the purpose or implications of actions [25]. Instrumental goals can be achieved in order to reach ultimate goals [26]. Unlike instrumental goals, ultimate life goals locate concrete situations in a person’s mental and behavioral framework that forms the core of self-identity [24]. They are irreplaceable in that they give meaning to our lives and without them our lives become meaningless [27]. However, in the course of one’s life, goals that give meaning may change. A reconstruction of the ultimate life goals in confrontation with contingency could assist patients to (re)access their own resources and come to terms with the unexpected aspects of life, ultimately improving their quality of life [28-30].

## Methods/Design

This study primarily aims to answer the following question: does an assisted structured reflection on life events and ultimate life goals of cancer patients improve quality of life? To evaluate the effect of the structured reflection we will conduct a multicenter two-armed randomized non-blinded controlled trial. Previous randomized studies on spiritual interventions in cancer patients have included patients from hospices or palliative care units [31-34]. However, spirituality is not restricted to end of life [35]. Therefore, in this study we will include patients who have been confronted with advanced cancer, but still have a life expectancy of at least half a year. The following inclusion and exclusion criteria apply:

Inclusion criteria

1. Patients ≥ 18 years of age with advanced cancer not amenable to curative treatment.

2. Life expectancy ≥ 6 months.

Exclusion criteria

1. Karnofsky Performance Score < 60.

2. Insufficient command of the Dutch language to fill out Dutch questionnaires.

3. Current psychiatric disease.

**Figure 1:**
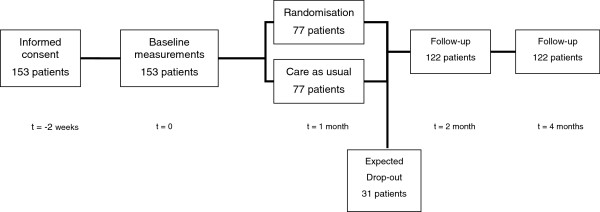
Study flow-chart.

Eligible patients will be invited by their treating oncologists and asked to give written informed consent. A baseline assessment will take place in consenting patients, including an evaluation of quality of life and spiritual wellbeing. Within two weeks after the baseline assessments patients will be randomized between an intervention and a control group (care as usual) (Figure 1).

Two months and four months after randomization, patients of both the intervention and the control group will complete questionnaires regarding quality of life, spiritual well being, empowerment and health care consumption. In the intervention group we will also conduct a telephone evaluation to examine the satisfaction with the intervention.

Patients declining participation will be asked to answer a few questions by telephone, to explore whether participants and non-participants differ.

### Intervention

We have developed a semi-structured interview model for the interpretation of contingent life events in life stories, based on literature on the experiences of contingency and the importance of ultimate life goals. In this interview we inquire into (a) the life events, (b) ultimate life goals, (c) the interpretation of contingent life events (d) reconstruction of life story. Since 2008 this interview model has been used in various populations, including mentally handicapped people, young people with problem behavior, individuals > 30 years, highly qualified young people in their twenties, Zen meditation trainees, volunteers in hospices, cancer patients, primary school teachers, and asylum seekers [24]. The respondents were from a religious and non-religious background. In all populations, the interviews were evaluated positively by most of the respondents. They did not experience the semi-structured nature of the interview as a drawback and frequently indicated that it was a very special experience to reconstruct their life stories in collaboration with an interviewer. In the experimental arm of our randomized study we will use this interview model for an assisted, structured reflection on contingent life events and ultimate life goals, which will be supported by a newly developed e-application. The assisted reflection is carried out in two consultations with a spiritual counsellor. The counsellor analyses the first consultation in the interim and discusses this analysis with the patient during the second consultation (Table 1).

**Table 1:** Summary of the intervention

**Consultation I**	**Analysis**	**Consultation II**
**Patient and spiritual counsellor**	**Spiritual counsellor**	**Patient and spiritual counsellor**
- Draw a life line	- Analyse life line	- Reflect on life line
- Explicate the most important events	- Analyse important life events	- Reflect on most important events
- Define the most important events	- Analyse life goals	- Reflect on life goals
- Draw a life line for the future	- Define coherence and tension between life goals and life events	- Discuss and reflect on tension and coherence between life goals and life events
- Define life goals		- Reconstruct life story

#### Consultation I

**Figure 2:**
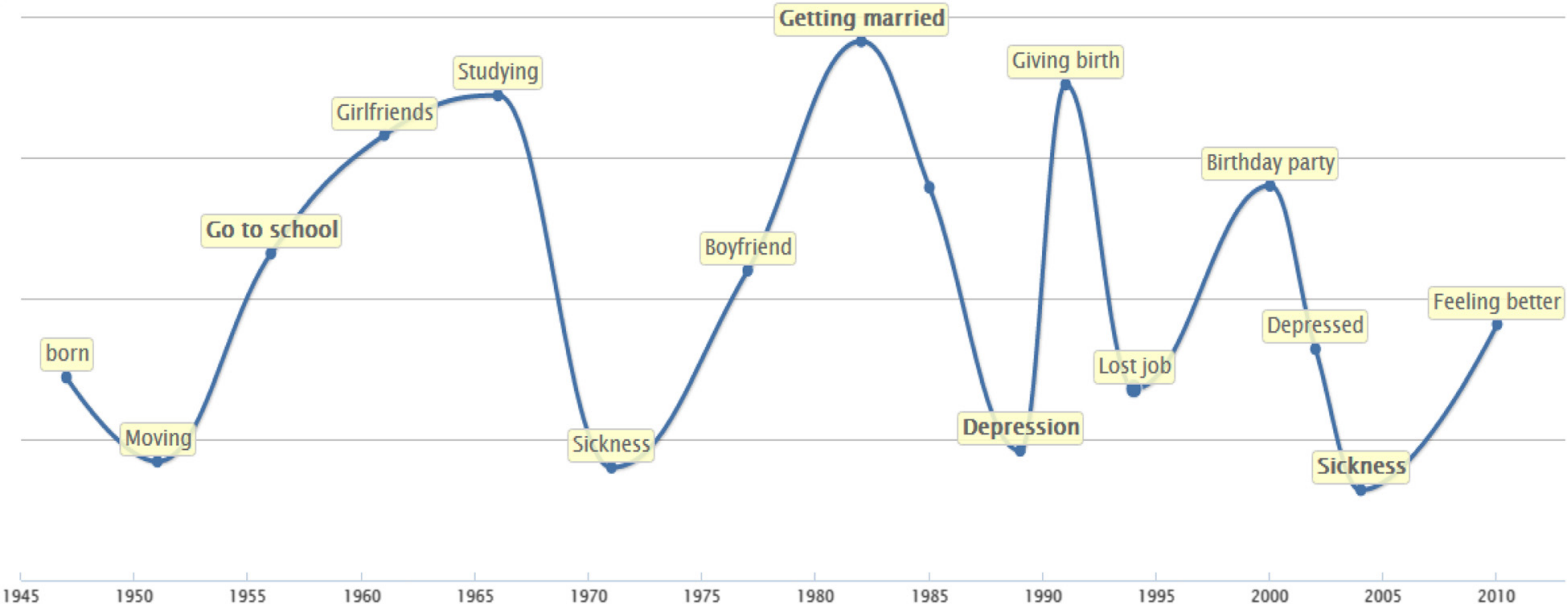
**Life line drawn using the e-application.** Looking back at their lives, patients indicate heights and lows.

In the first consultation with the spiritual counsellor, the patients draw their lifelines (Figure 2). The patients choose from their life line the three or four most important events and discuss these events with the counsellor. Next the patients draw their future life line and define life goals. In this first consultation, the spiritual counsellors use the interview model with specified questions in a given order. The interview model requires a probing technique, which implies that the spiritual counsellor keeps asking questions to unravel aspects of ultimate life goals as well as different layers in the interpretation of life events. The result of consultation I is a reconstruction of the patient’s life story and the reflection of the patients on this story.

#### Analysis of consultation I

**Figure 3:**
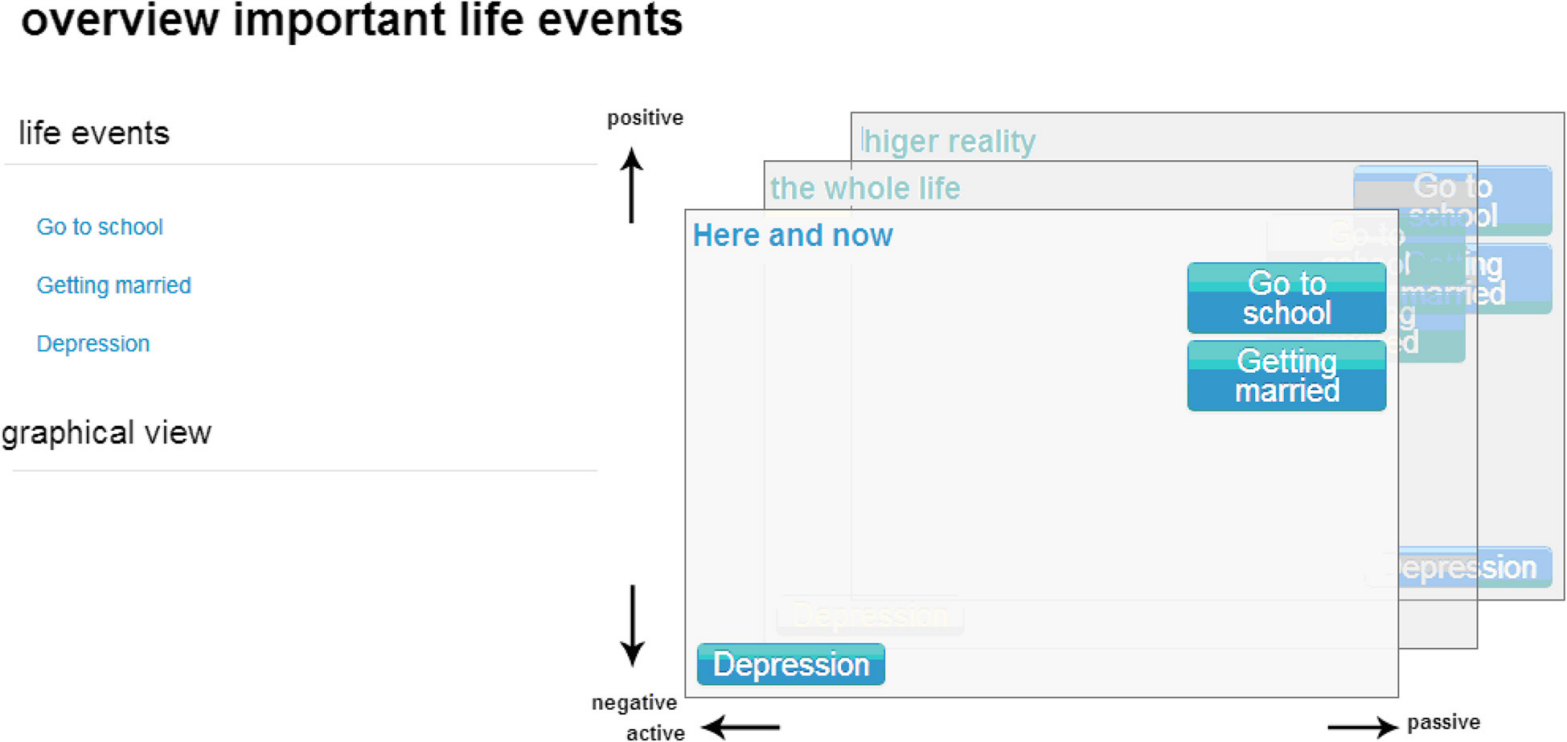
Classification of life events using the e-application.

The analysis of consultation I is performed by the spiritual counsellor and concerns three steps. First, using the e-application, the spiritual counsellor classifies the most important life events identified by the patient as active or passive and positive or negative. An active interpretation implies that the person views the event as an active effort in order to reach his/her own goals. A passive interpretation implies that the event happened to the person in a sense that something befalls you. A positive event means that the event foster's you in your striving to achieve a goal. Negative implies that the event hinders you in your striving to achieve a goal. The positive and negative interpretations relate to three dimensions of human thought and action [24]. These three dimensions are: ‘here and now’, ‘whole life’ and ‘a higher reality’ (Figure 3). Here and now implies that the event is situational; it has an impact on the person in the concrete situation. Whole life implies that the event is existential; it transcends the situational meaning and has an effect on the person's whole existence in time and space. Higher reality implies that the event is transcendental; it transcends the situational and existential meaning and has an effect on the person and his whole view of life.

**Figure 4:**
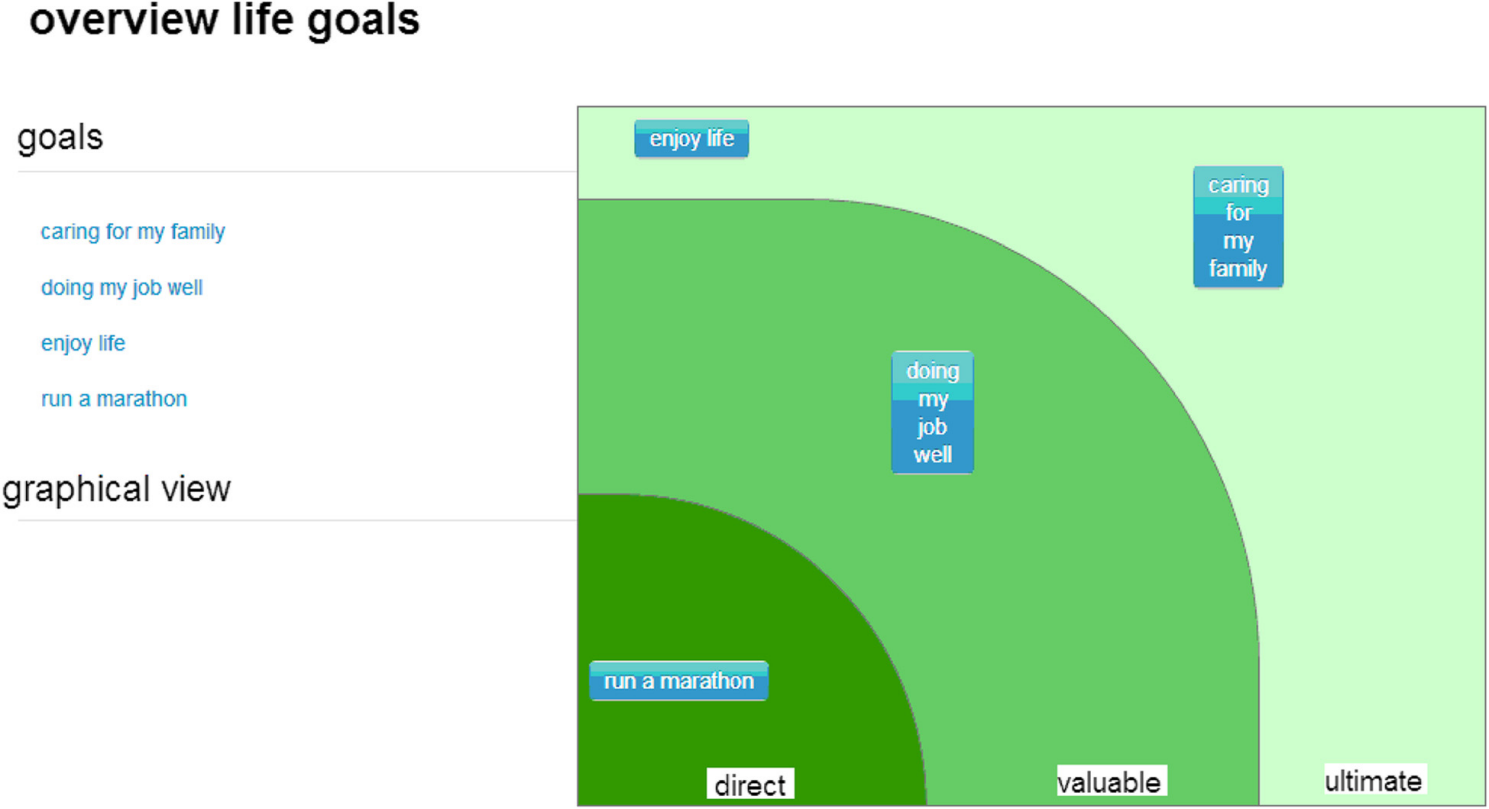
**Visual representation of life goals.** The five most important life goals identified by the patient are categorized as direct goals, valuable goals and ultimate goals.

**Figure 5:**
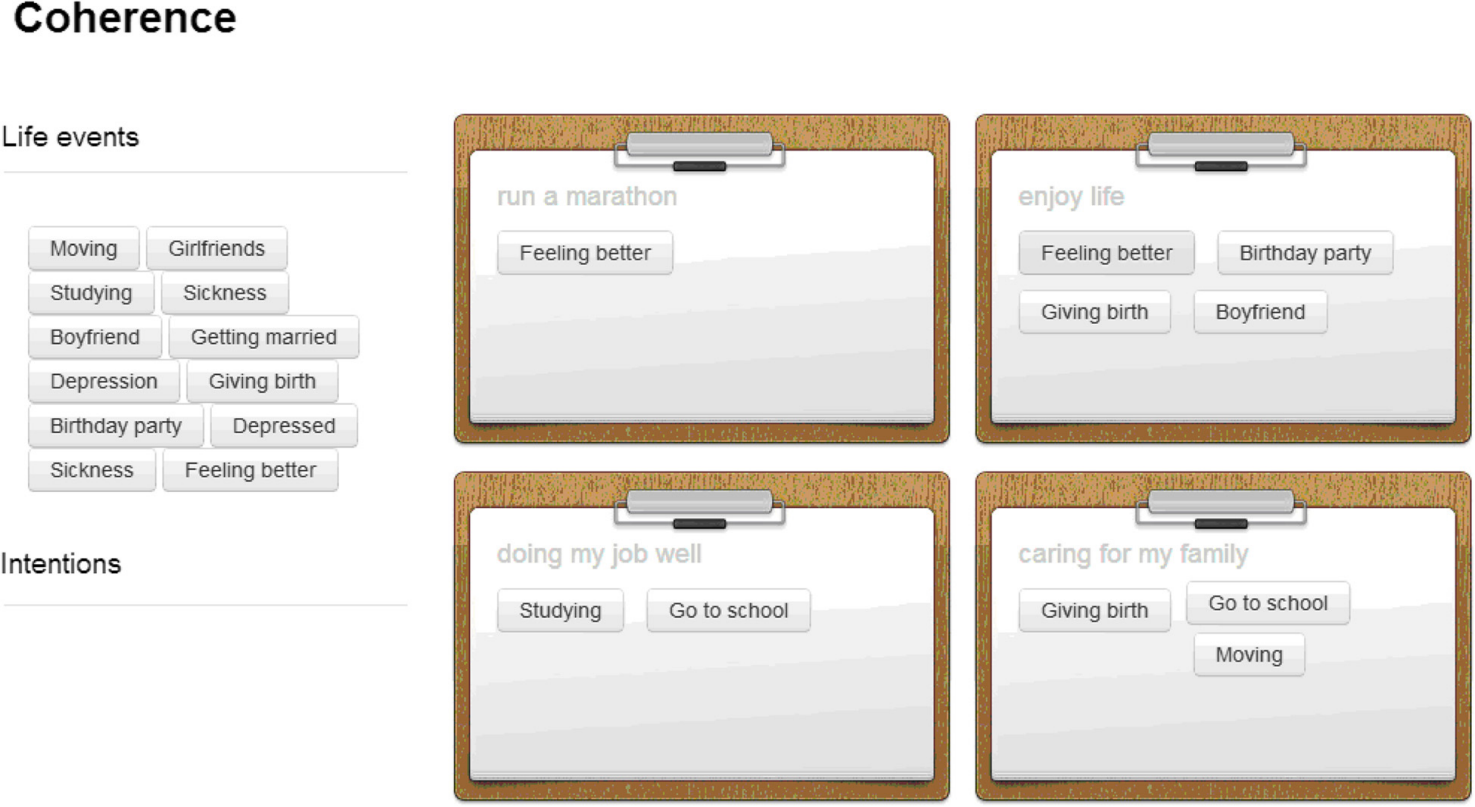
Identification of coherence and non-coherence between life events and life goals.

Second, the life goals that the patient defined in the first consultation are being weighed. Three different dimensions are taken into account: pre-intentional, intentional and meta-intentional [36]. The different dimensions help distinguishing between instrumental and ultimate life goals. The pre-intentional dimension describes instrumental life goals and comprises simple intentional ad hoc decisions such as eating when you are hungry. The intentional dimension describes more awareness for the good and evil in the environment. Finally, the meta-intention stage is where people define very abstract possibilities to transcend the world they are living in [24]. This results in a distinction between direct goals, valuable goals and ultimate goals (Figure 4). In the third and last step of the analysis the coherence between life goals and life events is indicated by the spiritual counsellor (Figure 5). The result of this whole analysis is a framework for observation and interpretation of contingent life events and ultimate life goals.

#### Consultation II

Using the analysis of Consultation I, the spiritual counsellor will summarize the results and present them to the patient in a transparent and organized way. The patient is thus aided during a one-hour session to reflect on his/her own framework for interpretation on a more profound level. Methodologically, this may be regarded as a member-check. However, at the same time the patient will be challenged to creatively respond to the results. The last screen on the e-application is built to be changed by the patients themselves. They discuss with the spiritual counsellor what kind of tension or coherence between life events and life goals can be identified (Figure 5). The patients are challenged to search for (in)coherence in their lives. This may aid patients accommodating their contingent life events [37].

### Endpoints

#### Primary endpoints

Two primary endpoints are distinguished. First, general quality of life as measured with the general quality of life scale of the European Organization for Research and Treatment of Cancer Quality of Life Questionnaire-Core 15 Palliative Care (EORTC QLQ-C15-PAL). The EORTC QLQ-C15-PAL is a shortened version of the EORTC QLQ-C30, which is one of the most rigorously studied and widely used health-related quality-of-life questionnaires in oncology research [38-41].

Second, spiritual wellbeing as measured by the subscale meaning/peace of the Functional Assessment of Chronic Illness Therapy - Spiritual wellbeing 12 (FACIT-Sp12). The FACIT-Sp-12 is a widely used measure and is not restricted to a particular religion and is valid and reliable [42]. The FACIT-sp-12 demonstrates good internal consistency reliability and a significant relation with quality of life in a large, multiethnic sample [43,44].

#### Secondary endpoints

Specific aspects of quality of life, as measured with the physical functioning and role functioning, and symptom scales of the EORTC QLQ-C15-PAL and the Faith subscale of the FACIT-Sp-12 will be treated as secondary endpoints.

Patient empowerment is becoming more and more important, both from health care professionals’ and from patients’ perspective [45]. Reconstructing a life story and also defining life goals and intention for the future can lead to a feeling of empowerment to undertake actions which are important. We will assess patients’ empowerment with a Dutch version of the Pearlin Mastery Scale developed by Pearlin en Schooler (1978) [46]. The Pearlin Mastery Scale measures the extent to which individuals perceive themselves in control of forces that significantly impact their lives. It consists of a 7-item scale. In previous studies, the instrument yielded satisfactory psychometric properties [47,48].

Furthermore, as patients’ view on spirituality can change over time as a result of the intervention, we will measure spirituality by the Spiritual Attitude en Interests List (SAIL), developed by the Helen Dowling Institute in the Netherlands. The SAIL is a multidimensional questionnaire for studying spiritual experiences of religious and nonreligious people with good internal consistency reliability [43].

#### Tertiary endpoints

Changes in patients’ perspective on satisfaction with life will be measured by the Diener Satisfaction with Life Scale [49]. Furthermore, as feelings of anxiety and depression may arise when patients realize the limited amount of time that is left to achieve life goals, feelings of anxiety and depression will be measured by the Hospital Anxiety and Depression Scale [50]. Also, patients’ health consumption is assessed according to a shortened and for this study adapted version of the Trimbos/iMTA questionnaire for Costs associated with Psychiatric Illness (TICP) [51]. Finally, we will explore patients’ satisfaction with the intervention by a telephone interview using a study-specific topic list.

#### Background variables

Demographic data, including data on religious/spiritual background, images of God and aspects of religious salience, as well as medical data, including tumor type, time since diagnosis and previous treatments, will be collected at baseline [52].

### Sample size calculation

The primary aim of our study is to improve quality of life and spiritual wellbeing. We will conduct a mixed design measures ANOVA to detect differences between the control-group and the intervention-group over pre-, post- and follow-up measurement. To detect a small effect (effect size f = .10) with statistical power 80%, alpha 5%, and a correlation between repeated assessments of r = .63, we need a sample of 122 patients. With an expected drop out of 20%, we will include 153 patients.

### Randomization

Randomization will be performed on-line via a secure internet facility in a 1:1 ratio by the TENALEA Clinical Trial Data Management System using randomly permuted blocks with maximum block size 4 within strata formed by nine spiritual counsellors. The researcher contacts the randomization website after patients have signed informed consent. The researcher enters the patient into the randomization program linked to the spiritual counsellor of the patients' hospital. In case in a specific hospital more than one spiritual counsellor is involved in the study a counsellor from that hospital is randomly allocated to the patient. Then the researcher receives the random treatment allocation (intervention versus control) for the patient.

### Recruitment

Seven hospitals accepted the invitation to join the study. Participating hospitals are two academic hospitals: the Academic Medical Centre in Amsterdam, University Medical Centre Utrecht in Utrecht. One categorical hospital: Antoni van Leeuwenhoek Ziekenhuis, and four local hospitals: Onze Lieve Vrouwe Gasthuis in Amsterdam, Elkerliek Ziekenhuis in Helmond, Westfriesgasthuis in Hoorn, and Spaarneziekenhuis in Hoofddorp.

### Ethical and legal considerations

The Medical Ethics Review Committee of the Academic Medical Centre Amsterdam confirmed that the Medical Research Involving Human Subjects Act (WMO) does not apply to our study and therefore an official approval of this study by the committee was not required. (Letter, June, 27^th,^, 2012)

### Sponsorship

This study is funded by The Dutch Cancer Society/Alpe d’HuZes and Janssen Pharmaceutical Companies.

## Discussion

This is the first randomized controlled trial to evaluate the role of an assisted structured reflection on life events and ultimate life goals to improve patients’ quality of life and spiritual wellbeing. Insight into one’s ultimate life goals is expected to help patients to integrate a life event such as cancer into their lives. A prospective study in patients is needed to empirically examine whether insight into one’s ultimate life goals improves quality of life and spiritual wellbeing. Since the intervention is brief and based on concepts and skills that spiritual counsellors are familiar with, it can be easily implemented in usual patient care and incorporated in guidelines on spiritual care [2].

Although we expect to find a positive outcome of our intervention on quality of life and spiritual wellbeing, we do realize that negative experiences may also be induced. For example, patients can become anxious or depressed when they bring life events from the past back into their memories [53,54]. We believe it is of utmost importance to assess the effects of our intervention therefore we will also measure for anxiety and depression.

Health care can benefit from technical innovations [55]. In our study we will use an e-application to support the analyses of the spiritual counsellor in a visually attractive way. The e-application will help obtaining a clearer view of the consultations’ content. Afterwards when patients receive the second questionnaire they also receive a printed version of the counsellor’s analysis. This printed version gives patients the opportunity to continue reflecting on their lifelines, interpretations of life events, life goals and the coherence between this all. Additionally, family and friends can have a look at this summary and discuss the results together, which may be of further benefit to the patients and their families.

As a result of this study, spiritual counsellors may be become more structurally involved in the health care of cancer patients. Referral to spiritual counsellors is already explicitly included in guidelines such as the NCCN guideline on distress [1]. However, in clinical practice only few spiritual counsellors are an integral part of the clinical team. We believe that evidence-based interventions on spiritual care will further improve the professionalization of spiritual counselling and structural incorporation into daily patient care.

Potential limitations of our study can be identified. The success of this study critically depends on the skills of the spiritual counsellors participating in the trial. However, spiritual counsellors involved in the study will all be experienced in patient care and will be trained to work with the interview model and e-application. This study will be conducted as a multicentre study, involving academic as well as peripheral hospitals. Therefore, we expect the generalizability of our results to be high. Nevertheless, generalizability will be limited by the national context of the study. In conclusion, by the conduction of this randomized controlled trial we aim to show the effectiveness oft a brief intervention that addresses spiritual concerns of cancer patients to improve quality of life.

## Competing interests

The authors declare that they have no competing interests.

## Authors’ contributions

HWMvL, MSR, MAGS, and JBAMS designed the study. RK, HWMvL, MSR, MAGS and JBAMS participate in the performance/conductance of the study. All authors critically reviewed the manuscript and approved the final version of the manuscript.

## Authors’ information

R. Kruizinga, MA and Dr. Hanneke W.M. van Laarhoven, MD, PhD are from Medical Oncology, Academic Medical Center. Prof. dr. M.A.G. Sprangers is from Medical Psychology, Academic Medical Center. Dr. Michael Scherer-Rath and Prof. Dr. J.B.A.M. Schilderman are from the Faculty of Philosophy, Theology and Religious Studies, Radboud University Nijmegen.

## Pre-publication history

The pre-publication history for this paper can be accessed here:

http://www.biomedcentral.com/1471-2407/13/360/prepub
